# COVID-19: Prevention and control measures in community

**DOI:** 10.3906/sag-2004-146

**Published:** 2020-04-21

**Authors:** Rahmet GÜNER, İmran HASANOĞLU, Firdevs AKTAŞ

**Affiliations:** 1 Department of Infectious Diseases and Clinical Microbiology, Faculty of Medicine,Yıldırım Beyazıt University,Ankara City Hospital, Ankara Turkey; 2 COVID-19 Advisory Committee of the Ministry of Health of Turkey Turkey

**Keywords:** COVID-19, Turkey, prevention, quarantine, social distancing, community

## Abstract

On January 30, 2020, the WHO declared the COVID-19 outbreak a public health emergency of international concern and, in March 2020, began to characterize it as a pandemic in order to emphasize the gravity of the situation and urge all countries to take action in detecting infection and preventing spread. Unfortunately, there is no medication that has been approved by the FDA, gone through controlled studies and demonstrated an effect on the virus for this global pandemic. Although there are cures for illnesses and developments made by leaps and bounds in our day, the strongest and most effective weapon that society has against this virus that is affecting not just health but also economics, politics, and social order, is the prevention of its spread. The main points in preventing the spread in society are hand hygiene, social distancing and quarantine. With increased testing capacity, detecting more COVID-19 positive patients in the community will also enable the reduction of secondary cases with stricter quarantine rules.

## 1. Introduction

In late 2019, a novel coronavirus, now designated SARS-CoV-2, was identified as the cause of an outbreak of acute respiratory illness in Wuhan, a city in the Hubei province of China. In February 2020, the World Health Organization (WHO) designated the disease COVID-19, which stands for coronavirus disease 2019. The clinical presentation of 2019-nCoV infection ranges from asymptomatic to very severe pneumonia with acute respiratory distress syndrome, septic shock and multi-organ failure, which may result in death [1]. On January 30, 2020, the WHO declared the COVID-19 outbreak a public health emergency of international concern and, in March 2020, began to characterize it as a pandemic in order to emphasize the gravity of the situation and urge all countries to take action in detecting infection and preventing spread.

The virus that causes COVID-19 is thought to spread mainly from person to person, mainly through respiratory droplets produced when an infected person coughs or sneezes. These droplets can land in the mouths or noses of people who are nearby or possibly be inhaled into the lungs. Other routes have also been implicated in the transmission of coronaviruses, such as contact with contaminated fomites and inhalation of aerosols, produced during aerosol generating procedures. Transmission of SARS-CoV-2 from asymptomatic individuals (or individuals within the incubation period) has also been described. However, the extent to which this occurs remains unknown [2].

Unfortunately, there is no medication that has been approved by the FDA, gone through controlled studies and demonstrated an effect on the virus for this global pandemic. Although there are cures for illnesses and developments made by leaps and bounds in our day, the strongest and most effective weapon that society has against this virus that is effecting not just health but also economics, politics, and social order, is the prevention of its spread. The interim guidance published by the WHO on 7 March 2020, “Responding to community spread of COVID-19,” states that preventing COVID-19 from spreading is through the development of coordination mechanisms not just in health but in areas such as transportation, travel, commerce, finance, security and other sectors which encompasses the entirety of society [3]. 

Preventive measures are the current strategy to limit the spread of cases. Early screening, diagnosis, isolation, and treatment are necessary to prevent further spread. Preventive strategies are focused on the isolation of patients and careful infection control, including appropriate measures to be adopted during the diagnosis and the provision of clinical care to an infected patient. Important COVID-19 prevention and control measures in community are summarized in Table.

**Table 1:** COVID-19 prevention and control measures in community.

Quarantine	Other measures:
Voluntary quarantine(self-quarantine)	Avoiding crowding
Mandatory quarantine o Private residence o Hospital o Public institution o Others (cruise ships, etc)	Hand hygiene
	Isolation
	Personal protective equipment
	School measures/closures
	Social distancing
	Workplace measures/closures

**Figure 1:**
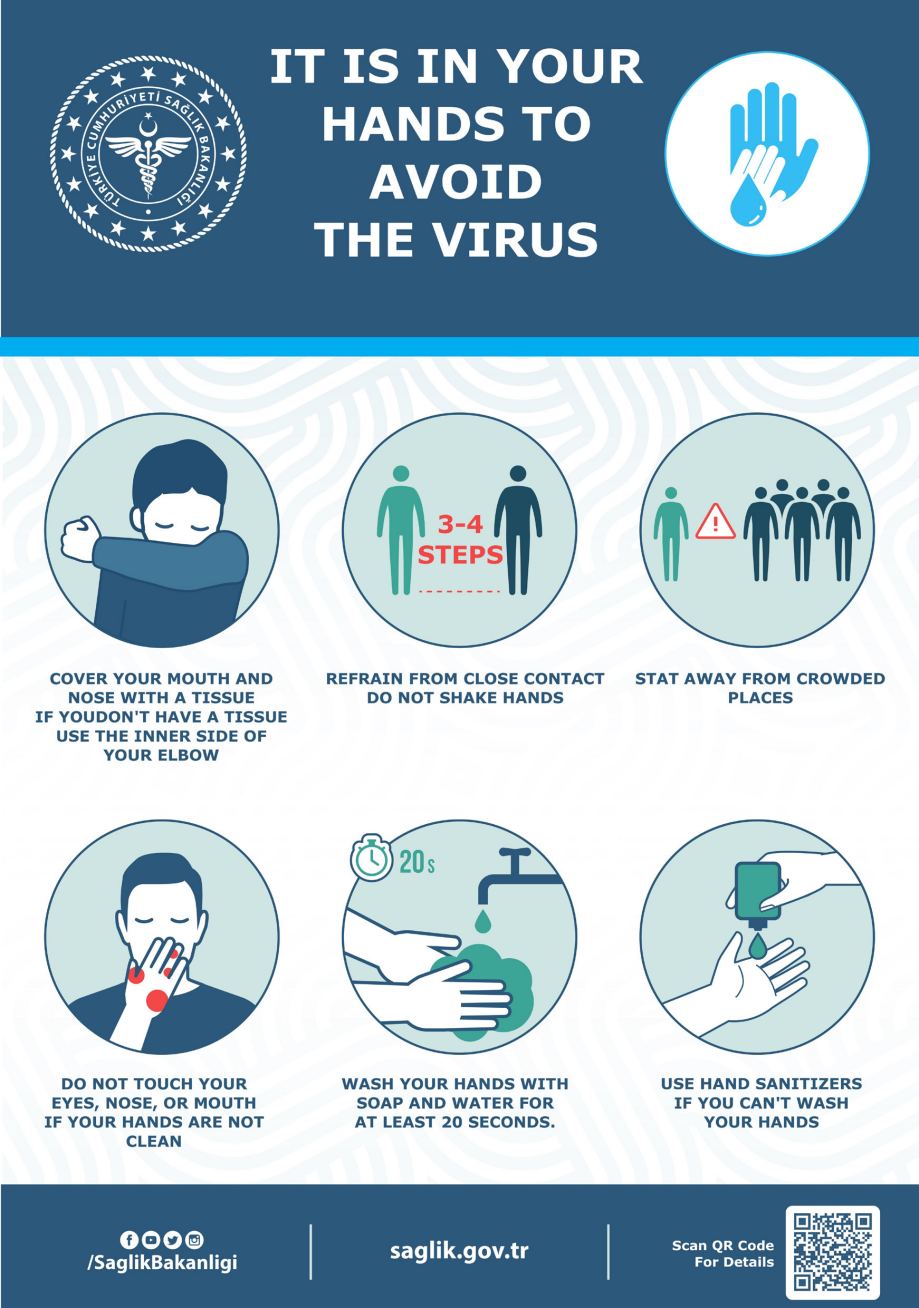
Poster regarding important prevention measures for COVID-19, prepared by Turkish Ministry of Health.

**Figure 2:**
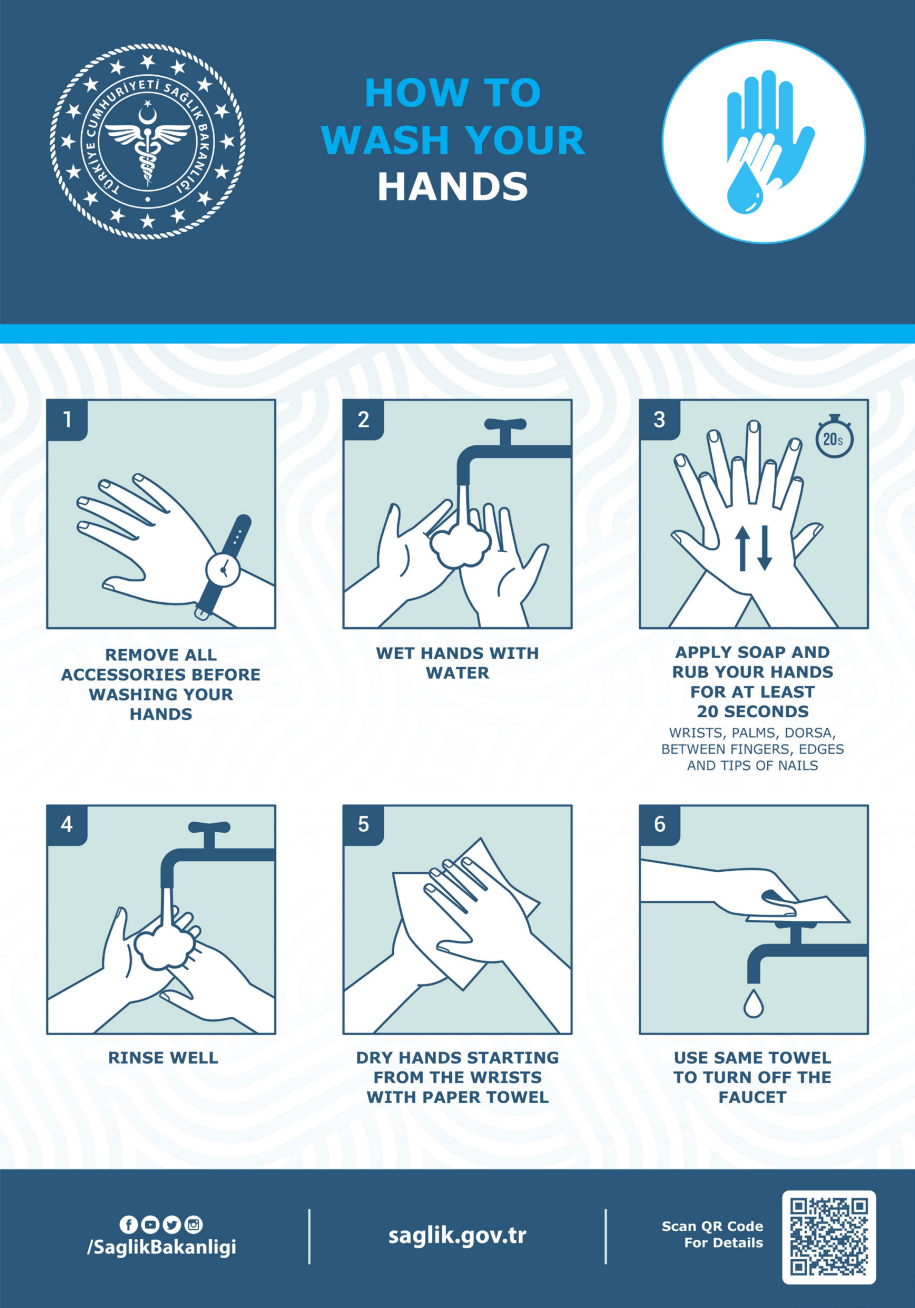
Poster regarding hand washing, prepared by Turkish Ministry of Health.

The most important strategy for the population to undertake is to frequently wash their hands and use portable hand sanitizer and avoid contact with their face and mouth after interacting with a possibly contaminated environment. To reduce the risk of transmission in the community, individuals should be advised to wash hands diligently, practice respiratory hygiene (i.e., cover their cough), and avoid crowds and close contact with ill individuals, if possible. There are posters and brochures prepared by many organizations on all issues related to protection from COVID-19 and are widely used all over the world (Figure 1).The WHO and other similar health organizations have published visual tools such as videos and posters to demonstrate the correct application of hand hygiene throughout the entire society (Figure 2).These posters, distributed throughout different parts of society in order to draw maximum attention to the importance of hand hygiene, created awareness among all of them. With the increase in the number of people carrying hand sanitizer with them for the application of instant hand hygiene and the spread of mask usage among people in countries such as China, Korea, and Japan, the pandemic was brought under control much more quickly. In those countries where such measures were not made mandatory, the exponential rise in the number of cases continues. 

Social distancing is advised, particularly in locations that have community transmission. Many countries have installed quarantine and social/physical distancing as measures to prevent the further spread of the virus.

These measures can include:

· The full or partial closure of educational institutions and workplaces,

· Limiting the number of visitors and limiting the contact between the residents of confined settings, such as long-term care facilities and prisons,

· Cancellation, prohibition and restriction of mass gatherings and smaller meetings,

· Mandatory quarantine of buildings or residential areas,

· Internal or external border closures, and

· Stay-at-home restrictions for entire regions or countries.

**Personal protective equipment**

For people without respiratory symptoms, the WHO does not recommend wearing a medical mask in the community, since it does not decrease the importance of other general measures to prevent infection. The single use of a mask does not obstruct the disease; the improper use of the mask actually increases the risk of COVID-19 infection. In the WHO’s “Advice on the use of masks in the context of COVID-19” interim guidance, the prioritized use of medical masks by health personnel was emphasized [4]. 

To reduce COVID-19 transmission from potentially asymptomatic or presymptomatic people, the ECDC recommends the use of face masks [5]. The use of face masks in the community may primarily serve as a means of source control. This measure can be particularly relevant in epidemic situations when the number of asymptomatic but infectious persons in the community can be assumed to be high. Wearing a face mask could be considered, especially when visiting busy, closed spaces, such as grocery stores, shopping centres, etc.; when using public transport; and for certain workplaces and professions that involve physical proximity to many other people (such as members of the police force, cashiers – if not behind a glass partition, etc.) and when teleworking is not possible.

In the United States, the CDC updated its recommendations in early April to advise individuals to wear a cloth face covering (i.e., homemade masks or bandanas) when in public settings where social distancing is difficult to achieve, especially in areas with substantial community transmission [6]. Individuals should be counseled to avoid touching the eyes, nose, and mouth when removing the covering, practice hand hygiene after handling it, and launder it routinely. 

The rationale for the face covering is primarily to contain secretions of and prevent transmission from individuals who have asymptomatic or presymptomatic infection. The CDC also reiterates that the face covering recommendation does not include medical masks, which should be reserved for health care workers.

Individuals who are caring for patients with suspected or documented COVID-19 at home should also wear a face cover when in the same room as that patient (if the patient cannot wear a face cover).

**Social distancing**

Social distancing is designed to reduce interactions between people in a broader community, in which individuals may be infectious but have not yet been identified hence not yet isolated [7]. As diseases transmitted by respiratory droplets require a certain proximity of people, social distancing of persons will reduce transmission. Social distancing is particularly useful in settings where community transmission is believed to have occurred, but where the linkages between cases is unclear, and where restrictions placed only on persons known to have been exposed is considered insufficient to prevent further transmission. Examples for social distancing include closure of schools or office buildings and suspension of public markets, and cancellation of gatherings. In public markets where it is difficult to maintain social distance, limitation of the entered person and encouraging online shopping can reduce the amount of contact.

Workplaces are also one of the high-risk areas for COVID-19 transmission. Therefore, home office working must be encouraged if possible. In workplaces where home office working is not possible, adherence to recommendations of WHO remains quite important [8].

Studies have been conducted that support the infectiousness of SARS-CoV-2 in the presymptomatic stage; social distancing is thus of critical importance in establishing control over the pandemic[2].

**Quarantine **

Quarantine is one of the oldest and most effective tools of controlling communicable disease outbreaks. This public health practice was used widely in fourteenth century Italy, when ships arriving at the Venice port from plague-infected ports had to anchor and wait for 40 days (in Italian: quaranta for 40) before disembarking their surviving passengers. The quarantine of persons is the restriction of activities of or the separation of persons who are not ill but who may been exposed to an infectious agent or disease, with the objective of monitoring their symptoms and ensuring the early detection of cases. Quarantine is different from isolation, which is the separation of ill or infected persons from others to prevent the spread of infection or contamination. 

Looking at the available studies in the literature, quarantine is the most effective method in reducing both the number of infected and dead [9,10]. It has been much more effective in countries which initiated strict quarantine rules right from the beginning. In an article quickly published by the Cochrane Library evaluating 29 studies, results indicate that quarantine can reduce the number of infected at rates from 81% to 44%, and in the number of dead from 61% to 31% [11]. 

In a mathematical model done on the spread of COVID-19 in Italy, it was shown that without strict quarantine rules the pandemic could not be controlled and that the number of secondary cases increased in proportion to the size of households. According to the simulation, if the household is comprised of 2 people and full quarantine has been put in place, expected secondary cases are 3 within the 14-day period; with a household of 6, this number increases to 16 [12]. 

Despite more than 2 months passing after the discovery of the first case in the US, the calls to stay at home put out in 33 states and by many local governments were insufficient. On the other hand, while it was greatly criticized, the quarantine and severe rules applied by China’s central government to people from Wuhan meant that they were able to effectively control the number of cases in states outside of Hubei and that death rates were reduced. 

In the influenza pandemic in 1918, the importance of quarantine measures was demonstrated very clearly [13]. The most striking example of this comes from the US–the first case in the city of Philadelphia, Pennsylvania, was observed on September 17, but social restrictions to prevent spread such as reducing crowds in public spaces were instituted on October 3, when there were 40 deaths per every 100,000 people. Unfortunately, the measures instituted after this point were insufficient and by the middle of October, this number reached 250/100,000 people. In contrast, the first case in St. Louis, Missouri, was observed on October 5, social restrictions were instituted on October 7, and both the number of cases and the rate of mortality was kept at low numbers. 

The WHO recommends that contacts of patients with laboratory-confirmed COVID-19 be quarantined for 14 days from the last time they were exposed to the patient [14]. For the purpose of implementing quarantine, a contact is a person who is involved in any of the following from 2 days before and up to 14 days after the onset of symptoms in the patient: 

· Having face-to-face contact with a COVID-19 patient within 1 meter and for >15 min,

· Providing direct care for patients with COVID-19 disease without using proper personal protective equipment,

· Staying in the same close environment as a COVID-19 patient (including sharing a workplace, classroom or household or being at the same gathering) for any amount of time,

· Travelling in close proximity with (that is, within 1 m separation from) a COVID-19 patient in any kind of conveyance.

Active monitoring of people who are quarantined is one of the important points for controlling the epidemic in the society. There are several mandatory mobile phone applications that control the compliance of people to quarantine in countries such as China, Japan and Korea. In Turkey, with the support of mobile phone operators, all persons who are quarantined are alerted instantly when they move away from their location. Certainly, deterrent fines will also increase compliance with quarantine.

**Cleaning and disinfection **

High-touch areas such as bedside tables and door handles should be disinfected daily with regular household disinfectant containing a diluted bleach solution (that is, 1-part bleach to 99 parts water). For surfaces that cannot be cleaned with bleach, 70% ethanol can be used. Toilets and bathrooms should be cleaned and disinfected with a diluted bleach solution (one part bleach to 9 parts water to make a 0.5% sodium hypochlorite solution). Disposable gloves should be used when cleaning or handling surfaces, clothing, or linen soiled with body fluids. All used disposable contaminated items should be placed in a lined container before disposing of them with other household waste. Clothes, bed linens, and bath and hand towels should cleaned using regular laundry soap and water or machine washed at 60–90°C with common laundry detergent. Disposable gloves should be used when cleaning or handling surfaces, clothing, or linen soiled with body fluids. All used disposable contaminated items should be placed in a lined container before disposing of them with other household waste.

**Increasing testing capacity**

Another important point in preventing the spread of the disease throughout society is to increase the number of tests and thus pinpoint more cases, isolate them, and trace those who have been in contact. For this reason, increasing laboratories’ test capacity and developing new testing strategies are of utmost importance. Different methods such as rapid-testing kits, serologic methods and self-collected specimen tests are being used throughout the world to determine cases which in turn help adherence to isolation rules. 

In South Korea, which acted quickly to administer free-of-charge and extensive public testing for COVID-19, “drive through testing” was initiated for the first time [15]. The ease of its application, reduction in the number of people who applied to health centres, and the capacity to investigate more people in lesser time appears as a successful strategy. Similar applications based on this model are being instituted in Germany and other countries after South Korea. 

**Prevention and control measures in Turkey**

Several different containment measures were implemented by the Turkish government. These included social distancing, travel restrictions on visitors arriving from high-risk counties, quarantine for nationals returning from high-risk locations, and closure of schools and certain types of workplaces. The government declared on March 12th that all schools including universities were to be closed starting from March 16th.

Turkey put into place several measures to limit movement of people. Citizens 65 years old or older, patients with immune system deficiency, chronic lung disease, asthma, COPD, chronic cardiovascular disease, chronic renal disease, hypertension, chronic liver disease as well as users of drugs that disrupt the immune system were restricted from leaving their homes and using public transportation. 

**Figure 3:**
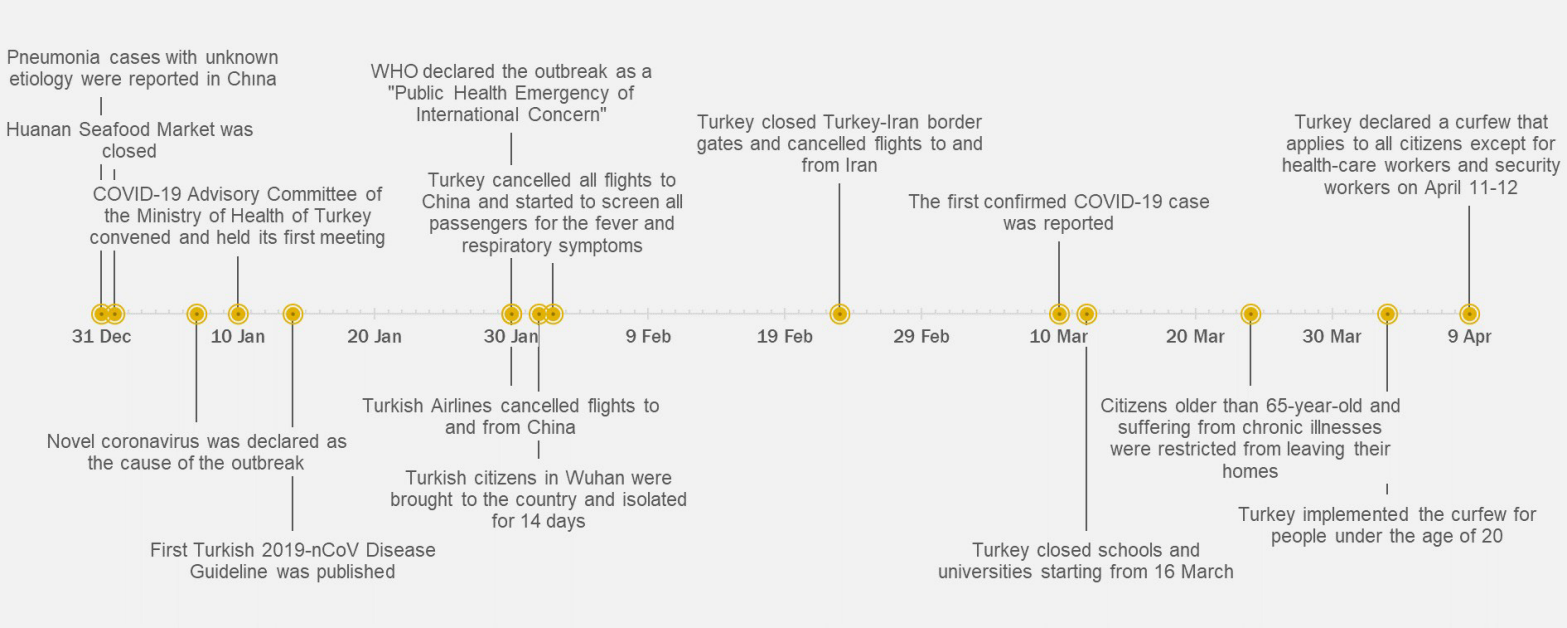
Timeline for prevention and control measures in Turkey.

Major containment actions taken are summarized in Figure 3. All ministries published general instructions on COVID-19 prevention and control measures in their organizations [16]. As of April 13, approximately 40,000 tests have been reached per day with a total of 73 authorized laboratories, and the number of performed daily tests is gradually increasing.

## 2. Conclusion

In COVID-19, which has no approved treatment, it is very important to prevent the spread in the society. The main points in preventing the spread in society are hand hygiene, social distancing and quarantine. With increased testing capacity, detecting more positive patients in the community will also enable the reduction of secondary cases with stricter quarantine rules.

## Acknowledgments

Rahmet GÜNER and Firdevs AKTAŞ are the members of the COVID-19 Advisory Committee of the Ministry of Health of Turkey. Rahmet GÜNER and İmran HASANOĞLU are working in the main pandemic hospital, Ankara City Hospital, a 3800-bed hospital with 700 ICU beds.

## References

[ref0] Clinical Characteristics of Coronavirus Disease 2019 in China. New England Journal of Medicine 2020 February 28. doi: 10.

[ref1] (2020). Presymptomatic Transmission of SARS-CoV-2 -. MMWR Morbidity and Mortality Weekly Report.

[ref2] (2020). Responding to community spread of COVID-19 [online]. Website https://www.who.int/ publications-detail/responding-to-community-spread-ofcovid-
19.

[ref3] (2020). Advice on the use of masks in the context of COVID-19: interim guidance, 6 April 2020 [online]. Website https://apps.who.int/iris/handle/10665/331693.

[ref4] (2020). Using face masks in the community reducing COVID-19 transmission from potentially asymptomatic or pre-symptomatic people through the use of face masks or presymptomatic people through the use of face masks ECDC Technical Report [online].. Website https://www.ecdc.europa.
eu/en/publications-data/using-face-masks-communityreducing-
covid-19-transmission.

[ref5] (2020). Centers for Disease Control and Prevention (CDC). Website https://www.cdc.gov/coronavirus/2019-ncov/prevent-gettingsick/
cloth-face-cover.html.

[ref6] (2020). Isolation, quarantine, social distancing and community containment: pivotal role for old-style public health measures in the novel coronavirus (2019-nCoV) outbreak. Journal of Travel Medicine.

[ref7] (2020). Getting your
workplace ready for COVID-19: How COVID-19 spreads, 19 March 2020 [online]. Website https://apps.who.int/iris/ handle/10665/331584.

[ref8] (2020). Association of Public Health Interventions With the Epidemiology of the COVID-19 Outbreak in Wuhan, China. Journal of the American Medical Association 2020 April.

[ref9] Why does Japan have so few cases of COVID19? EMBO Molecular Medicine 2020 April 10.

[ref10] Quarantine alone or in combination with other public health measures to control COVID-19: a rapid review. Cochrane Database Systematic Review 2020 April.

[ref11] (2020). Only strict quarantine measures can curb the coronavirus disease (COVID-19) outbreak. Eurosurveillance.

[ref12] (2007). Public health interventions and epidemic intensity during the 1918 influenza pandemic. Proceedings of the National Academy of Sciences of the United States of America.

[ref13] (2020). Considerations for quarantine of individuals in the context of containment for coronavirus disease (COVID-19) [online]. Website u2b41 [accessed 12 April.

[ref14] (2020). Drive-through screening center for COVID-19: a safe and efficient screening system against massive community outbreak. Journal of Korean Medical Sciences.

[ref15] (2020). T.C. Sağlık Bakanlığı (2020). Kurumlar İçin Alınan Kararlar [online].. Website https://covid19bilgi.saglik.gov.tr/tr/alinankaralar.htm.

